# Amputation in patients with extremity soft tissue sarcoma: the experience of an East Asian referral center

**DOI:** 10.1186/s12885-023-11813-2

**Published:** 2024-01-11

**Authors:** Yongsung Kim, Han-Soo Kim, Ilkyu Han

**Affiliations:** 1 Department of Orthopaedic Surgery, Seoul National University Hospital, 101 Daehak-ro Jongno- gu, 03080 Seoul, Korea; 2 Department of Orthopaedic Surgery, Seoul National University College of Medicine, 101 Daehak-ro Jongno-gu, 03080 Seoul, Korea; 3 Department of Orthopaedic Surgery, Seoul National University Bundang Hospital, 82, Gumi-ro 173 Beon-gil, Bundang-gu, Seongnam, Korea

## Abstract

**Background:**

This study aimed to investigate the characteristics and clinical outcomes in a series of patients with extremity soft tissue sarcoma (STS) who underwent amputation at a large East Asian referral center.

**Patients and methods:**

Of the 652 patients who underwent surgery for extremity STS, data of 37 consecutive patients who underwent amputation were reviewed retrospectively. The median follow-up period was 96.0 months (range, 15–216). The patients were classified in to three cohorts. The primary localized (PL) group included patients who underwent amputation as a primary surgical procedure with curative intent. The recurrent localized (RL) group included patients who underwent amputation as a revision procedure after failure of previous limb sparing surgeries. The metastatic group included patients who underwent amputation as a palliative procedure.

**Results:**

There were 22 cases of amputation in 596 STS patients and the amputation rate was 3.6% (22/596). Further, 1.8% (9/490) of patients with primary localized STS underwent amputation. Patients with localized STS who underwent amputation had a 5-year disease-specific survival (DSS) rate of 89.9% (95% Confidence Interval (CI), 87.1–92.7%), a local-recurrence-free survival (LRFS) of 84.1% (95% CI, 80.5–87.6%), and a metastasis-free survival (MFS) of 84.6%. (95% CI, 81.1–88.0%) Compared with previous studies, our results showed higher DSS and MFS rates with similar LRFS.

**Conclusions:**

The amputation rate of extremity STS in our institute in East Asia was similar but slightly lower than that reported in Western studies. The oncologic outcome of amputation reported in this study was higher than that indicated in Western studies and oncologic outcome of amputation was not statistically different from those of limb salvage surgery. However, considering the small cohort in single institute study, there is a possibility of selection bias and future multi-center study is necessary. From our results, amputation is still a feasible option for appropriately selected patients unsuitable for limb-conserving surgery.

## Introduction

Amputation had been the optimal treatment strategy for achieving local tumor control in patients with extremity soft tissue sarcoma (STS) until limb salvage surgery was introduced. The rationale of amputation for local tumor control was based on the results obtained from patients who underwent surgery alone [1–3]. Limb salvage surgery (LSS) was introduced in the 1970s, and many studies supporting this strategy were reported. Eilber et al. showed a 91% of excellent local control rate in bone and soft tissue sarcoma patients treated with limb salvage combined with chemotherapy and radiotherapy. In subgroup analysis, 5-year disease-specific survival (DSS) of 65 STS patients with AJCC stage I, grade 3, was 75% while those of patients treated by surgery alone which was obtained by the American Joint Committee of stage I, grade 3 tumors were 21% [1]. Radiation therapy is beneficial in local control after tumor resection and chemotherapy seems to contribute to improvement in DSS [4, 5]. Williard et al. emphasized the importance of adjuvant treatment and showed no difference in the overall survival and metastasis-free survival (MFS) between the LSS group and the amputation group [5, 6]. In addition, with advances in surgical techniques such as the reconstruction of major vessels and nerves and flap [7], LSS is considered as the standard treatments for extremity STS [8–10]. With advances in LSS, the rate of amputation decreased from 40 to 50% to < 10% [11–13]. With advances in techniques facilitating LSS, amputation is rarely performed in patients with extremity STS [2, 14]. Despite these advances in the local management of STS, certain indications show that amputation may be a better option for definite local management [15, 16]. According to literature, the current indications for amputation as local management of extremity STS are as follows: (1) anticipated inadequate limb function after R0 resection, (2) multi-compartmental neurovascular tumor involvement, and (3) local tumor contamination from previous unplanned surgery [16].

In East Asia, there are few studies on the rate of amputation. In a systematic review of STS in the Asia-Pacific region, the amputation rate was variable depending on countries from 4 ~ 31% [17]. A recent Japanese report of 12 hospitals of Tokai Musculoskeletal Oncology Consortium with 55 amputations showed excellent local control and acceptable functional outcomes that 60.9% of the patients could walk using artificial limbs [18]. Studies with the SEER database in the United States showed that the amputation rate of STS in Asians was lower than that in other races [19, 20]. Therefore, we aimed to review the data of patients who underwent amputation due to extremity STS in our institution and analyze the clinical outcome. In addition, we aimed to compare our results with those of previous literature from Western studies. Specifically, this study investigated (1) the amputation rate of our cohort, (2) the clinic-pathologic characteristics and indications in a series of extremity STS patients who underwent amputation at a single East Asian referral center, and (3) compared the oncologic outcomes including DSS, local recurrence-free survival (LRFS), and MFS between the amputation group and limb salvage group and (4) performed literature reviews.

## Materials and methods

### Patients’ demographics and clinic-pathologic characteristics

The study was approved by the Institutional Review Board (IRB No. H-1811-148-989). We retrospectively reviewed the medical records of 652 patients with extremity STS who underwent surgical treatment at our institution between 2000 and 2017. The patients were followed up for at least 1 year. Among them, 37 patients underwent amputation and were included in the final cohort.

The median follow-up period was 96.0 months (range, 15–216). All patients underwent multidisciplinary evaluation at our institution preoperatively. Staging studies included computed tomography of the chest and magnetic resonance imaging of the involved limb. The main attributable reason and indications for amputation in each patient in this series were categorized. The patients were classified in to three cohorts. The primary localized (PL) group included patients who underwent amputation as a primary surgical procedure with curative intent. These patients had no metastasis at the time of diagnosis and underwent amputation within a certain period (1 month) after biopsy. The recurrent localized (RL) group included patients who underwent amputation as a revision procedure after failure of previous limb sparing surgeries. The metastatic group included patients who underwent amputation as a palliative procedure. These patients had metastasis at the time or before amputation. On location, proximal amputations were defined as amputations performed proximity to the elbow joints in the upper extremities and knee joints in the lower extremities.

### Methods

Data of the three study groups were reviewed and compared according to the patient demographics, tumor characteristics, and adjuvant treatments. Demographic data included gender, age at diagnosis, surgical treatment, level of amputation, and oncological outcome. The tumor characteristics obtained from patients’ medical records included tumor size, tumor location, depth, histological subtype, postoperative histological grade, and postoperative pathologic margin. The indications for performing amputation and levels of amputation were discussed preoperatively during the multidisciplinary conference. The amputation rate of the entire cohort was determined by dividing the number of amputations for localized diseases by the number of limb salvage surgeries. The primary amputation rate was also assessed, in which only the number of amputations performed as primary procedures were considered. Lastly, our results were compared with those reported in Western literature.

### Statistical analysis

All oncological outcomes were defined as the time from the date of surgery to the occurrence of events. The DSS, LRFS, and MFS were assessed and plotted using the Kaplan-Meier method and the log-rank test. SPSS version 24.0 (IBM Corp., Armonk, NY, USA) was used to perform all analyses.

## Results

### Demographics

The median age of 37 patients who underwent amputation was 54 years (range, 19–75) (Table 1). The male-to-female ratio was 22:15, with the proportion of male patients being higher than that of female patients. Regarding the location, 46% (17 cases) of the tumors were in the upper extremities, and 54% (20 cases) were in the lower extremities. Approximately 96% (35 cases) of the tumors were deeply located. The median size of the tumors was 6.4 cm. (range, 1–26). Undifferentiated pleomorphic sarcoma (UPS) was the most common histology (32.4%, 12 cases), followed by myxofibrosarcoma and epithelioid sarcoma (13.5%, 5 cases each). In terms of postoperative histologic grade according to the Federation Nationale des Centers de Lutte Contre le Cancer grading system, 51.4% (19 cases) of the tumors were grade 3. In postoperative pathologic margins, 91.9% (34 cases) were negative margins. When the PL group (*n* = 9) was compared with the RL group (*n* = 13), no differences were observed in the median age of surgery (59 years). No significant difference was also observed among the patients in the PL group (men: 4, women: 5) in terms of gender, while men were dominant in the RL group (men: 9, women: 4). Lower extremities were the most common tumor location in the PL group (66.6%), while the upper extremities in the RL group (76.9%). The most common level of amputation was below the knee in the entire cohort. No differences were found in the tumor size between the two study groups. The metastatic group showed larger tumor size compared with the PL and RL groups. Moreover, myxofibrosarcoma and epithelioid sarcoma were the most common histologic subtypes in the PL group (22.2%), while UPS was in the RL group (46.2%). In terms of histologic grade, 44.4% of patients in the PL group had grade 3 tumors, while 61.5% of patients in the RL group had grade 2 tumors. All patients in the PL group had deep-seated tumors and negative surgical margins.

**Table 1:** Patient demographics and tumor characteristics

	Primary localized	Recurred localized	Metastatic	Overall
Number of patients	9	13	15	37 (100)
Median age at operation	59.0 (22–73)	59.0 (25–75)	39.0 (19–72)	54.0 (19–75)
Male: female ratio	0.80 (4:5)	2.25 (9:4)	1.46 (9:6)	1.46 (22:15)
Level of amputation				
Upper limb				
Total	3 (33.3)	10 (76.9)	4 (26.7)	17 (46.0)
Below elbow	1 (11.1)	4 (30.8)	0 (0)	5 (13.5)
Above elbow	1 (11.1)	5 (38.5)	3 (20.0))	9 (24.3)
Shoulder disarticulation	1 (11.1)	1 (7.7)	0 (0)	2 (5.4)
Forequarter	0 (0)	0 (0)	1 (6.7)	1 (2.7)
Lower limb				
Total	6 (66.6)	3 (23.1)	11 (73.3)	20 (54.0)
Below foot	2 (22.2)	0 (0)	0 (0)	2 (5.4)
Below knee	3 (33.3)	2 (15.4)	5 (33.3)	10 (27.0)
Above knee	1 (11.1)	1 (7.7)	5 (33.3)	7 (18.9)
Hindquarter	0 (0)	0 (0)	1 (6.7)	1 (2.7)
Median tumor size	4.80 (1–18)	4.85 (1–20)	7.00 (2–26)	6.40 (1–26)
Histological subtype				
Myxofibrosarcoma	2 (22.2)	3 (23.1)	0 (0)	5 (13.5)
Undifferentiated pleomorphic sarcoma	1 (11.1)	6 (46.2)	5 (33.3)	12 (32.4)
Liposarcoma	1 (11.1)	1 (7.7)	0 (0)	2 (5.4)
Synovial sarcoma	1 (11.1)	0 (0)	2 (13.3)	3 (8.1)
Leiomyosarcoma	0 (0)	1 (7.7)	0 (0)	1 (2.7)
Rhabdomyosarcoma	0 (0)	0 (0)	2 (13.3)	2 (5.4)
Epitheliod sarcoma	2 (22.2)	2 (15.4)	1 (6.7)	5 (13.5)
Clear cell sarcoma	0 (0)	0 (0)	3 (20.0)	3 (8.1)
Malignant hemangiopericytoma	1 (11.1)	0 (0)	0 (0)	1 (2.7)
Malignant peripheral nerve sheath tumor	1 (11.1)	0 (0)	0 (0)	1 (2.7)
Extraskeletal osteosarcoma	0 (0)	0 (0)	2 (13.3)	2 (5.4)
Histologic grade (FNCLCC)				
Grade 1	1 (11.1)	0 (0)	0 (0)	1 (2.7)
Grade 2	2 (22.2)	8 (61.5)	2 (13.3)	12 (32.4)
Grade 3	4 (44.4)	2 (15.4)	13 (86.7)	19 (51.4)
Unknown	2 (22.2)	3 (23.1)	0 (0)	5 (13.5)
Depth				
Deep	9 (100)	12 (92.3)	14 (93.3)	35 (94.6)
Superficial	0 (0)	1 (7.7)	1 (6.7)	2 (5.4)
Pathologic margin				
Negative margin	9 (100)	11 (84.6)	14 (93.3)	34 (91.9)
Positive margin	0 (0)	2 (15.4)	1 (6.7)	3 (8.1)
Adjuvant treatment				
Preoperative radiotherapy	0 (0)	4 (30.8)	0 (0)	4 (10.8)
Postoperative radiotherapy	1 (11.1)	7 (53.9)	5 (33.3)	13 (35.1)
Preoperative chemotherapy	1 (11.1)	1 (7.7)	0 (0)	2 (5.4)
Postoperative chemotherapy	2 (22.2)	2 (15.4)	7 (46.7)	11 (29.7)

Data are expressed as n(%) unless otherwise specified

FNCLCC, Federation Nationale des Centers de Lutte Contre le Cancer

### Amputation rate for localized disease

Of the 37 patients, 22 had localized STS and 15 had metastatic disease at the time of surgery. The amputation rate for localized STS was 3.6% (22/596). The primary amputation rate was 1.8% (9/490). These numbers were lower than those reported in Western studies. The amputation rate for localized disease and the primary amputation rate were the lowest among the values reported in studies on amputation published after 2000 (Table 2).

**Table 2:** References on amputation rate of extremity soft tissue sarcoma in Western studies

Author	Year	Number of cohort	Number of amputation	Amputation rate for localized disease (%)	Primary amputation rate (%)
Eliber et al.	1980	105	21	52.5	NA
Collin et al.	1987	107	34	31.7	NA
Williard et al.	1992	649	92	14.1	NA
Keus et al.	1994	156	13	8.3	NA
Pitcher et al.	1994	219	9	4.1	2.4
Pitcher et al.	2000	439	21	4.7	NA
Trovik et al.	2001	1613	NA	22	9.6
Ghert et al.	2005	413	25	6.3	6.3
Potter et al.	2009	170	26	15.3	NA
Alamanda et al.	2012	278	16	5.7	3.5
Stevenson et al.	2017	NA	39	NA	NA
Smith et al.	2018	556	69	10.6	4.1
Erstad et al.	2018	NA	54	NA	NA
Current study	2023	596	22	3.6	1.8

NA: not available

### Indications for amputation: primary localized vs. recurrent localized

The patients’ medical records were reviewed, and the treatment intent for amputation in the PL and RL groups were analyzed (Table 3). In the PL group, the average period from diagnosis to surgical treatment was less than a month, while that in the RL group was 117.5 months (range, 9–183, median, 21 months). The median number of limb salvage surgeries before amputation in the RL group was 4 (range, 1–13). With regard to the indications for amputation, 62% of the patients in the RL group and 11% of the patients in the PL group showed tumor multifocality (*p* = 0.031). Unplanned excision was performed in 44% of the patients in the PL group and 23% of the patients in the RL groups (*p* = 0.376). The proportions of patients who developed inevitable functional deficit after surgery and functional deficit due to tumor at the time of surgery were similar between the PL group (22%) and RL group (23%). (*p* = 1). Neurovascular involvement was also found in 100% of the patients in the PL group and 77% of the patients in the RL group. When the tumor location was divided into proximal and distal according to their distance from the elbow joint in the upper extremity and knee joint in the lower extremity, 22% (2 cases) of the tumors in the PL group located in the proximal part, whereas 53% (7 cases) of the tumors in the RL group were found in the proximal part (*p* = 0.203). Wound complications at the time of amputation developed in 31% of patients from the RL group and in 11% of the patients from the PL group (*p* = 0.36). No significant difference was observed in pain intensity, palpable mass, joint involvement, and multi-compartment involvement between the PL group and RL group.

**Table 3:** Characteristics of indication for amputation in localized amputation

Variable	Primary localized *n* = 9 (%)	Recurred localized *n* = 13 (%)	Overall *n* = 22 (%)	p-value	
Unplanned excision	4 (44)	3 (23)	7	0.376	
Inevitable functional deficit after excision	7 (78)	12 (92)	19	1	
Functional impairment due to tumor	2 (22)	3 (23)	5	1	
Wound complication	1 (11)	4 (31)	5	0.36	
Palpable mass	7 (78)	10 (77)	17	1	
Pain	7 (78)	9 (69)	16	0.156	
Joint involvement	7 (78)	8 (62)	15	0.333	
Neurovascular involvement	9 (100)	10 (77)	19	0.24	
Multifocal tumor	1 (11)	8 (62)	9	0.031*	
Multiple compartments involved	7 (78)	13 (100)	20	0.156	
Proximal location	2 (22)	7 (53)	9	0.203	

Data are expressed as n(%) unless otherwise specified

### Oncologic outcome of amputation with localized disease

**Fig. 1:**
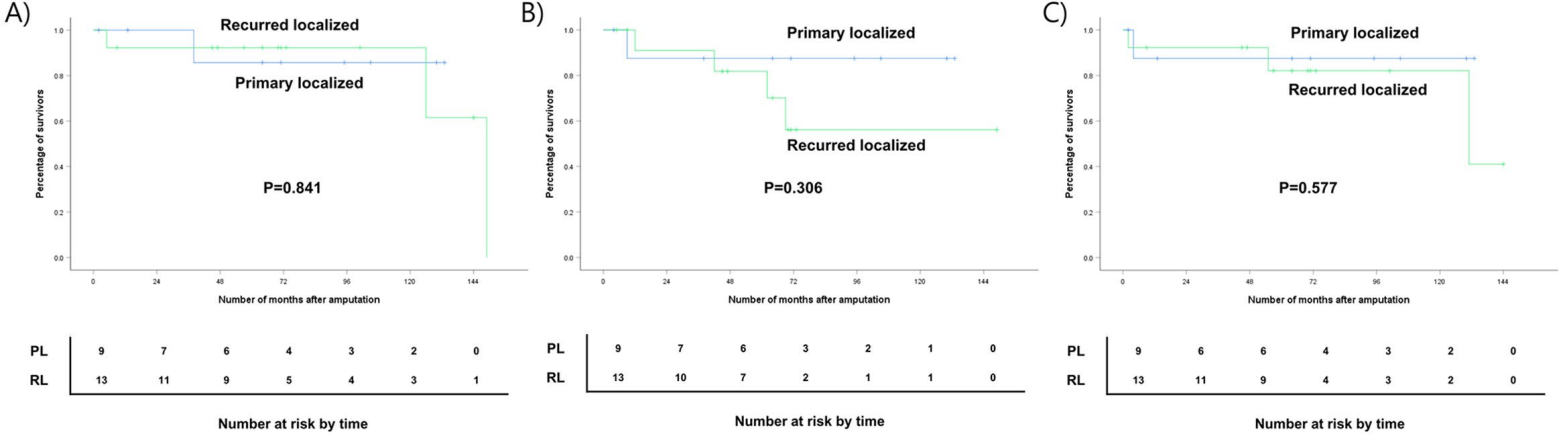
Survival analyses of amputation with localized diseases. (**A**) Disease-specific survival (*P* = 0.995), (**B**) Local recurrence-free survival (*P* = 0.503), and (**C**) Metastasis-free survival (*P* = 0.703) of primary localized group and recurrent localized group

In the entire cohort who underwent amputation for localized STS, the 5-year DSS was 89.9% (95% CI, 87.1–92.7%), the 5-year LRFS was 84.1% (95% CI, 80.5–87.6%), and the 5-year MFS was 84.6% (95% CI, 81.1–88.0%). Compared with previous studies, our results showed higher DSS and MFS rates with similar LRFS rate compared with the 3-year survival rates reported by Smith et al. (Table 4). This tendency was maintained in the subgroup analysis. The 5-year DSS of PL group and RL group were 85.7% (95% CI, 77.1–94.3%), and 92.3% (95% CI, 88.2–96.3%) (*p* = 0.841), respectively. The 5-year LRFS of both groups were 87.5% (95% CI, 79.8–95.1%) and 81.8% (95% CI, 77.1–86.5%) (*p* = 0.306) while the 5-year MFS were 87.5% (95% CI, 79.8–95.1%) and 82.1% (95% CI, 75.7–88.4%) (*p* = 0.577), respectively (Fig. 1). Both groups showed higher DSS and MFS rates and similar or slightly higher LRFS rates compared with those reported in studies conducted by Erstad et al., Smith et al., and Stevenson et al. (Table 4). In the entire cohort, four patients developed metastatic disease (18.8%), with a median time to first metastasis of 30 months. Only one patient in the PL group died after developing distant metastasis in the lungs, while two patients died in the RL group. The most common site of first metastasis was the lung, accounting for 3 out of 4 patients (75%). One patient developed chest wall metastasis.

**Table 4:** Comparison of oncologic outcomes with Western studies

		Current study	Erstad et al.	Smith et al.	Stevenson et al.
Period		2000–2017	2001–2011	2004–2014	1996–2016
Number of cohort (amputated)		37	54	69	39
Published		2023	2018	2018	2017
Amputation number and rate for localized STS		3.4% (22/596)	(*n* = 54)	(*n* = 59)	(*n* = 39)
Amputation number and rate for primary localized STS		1.8% (9/490)	(*n* = 18)	4.1% (23/556)	(*n* = 16)
Localized STS	DSS	89.9%	NA	49.8%(3 year)	NA
	LRFS	84.1%	NA	89.6%(3 year)	NA
	MFS	84.6%	NA	44.0%(3 year)	NA
Primary localized STS	DSS	85.7%	68%	30.5%(OS, 3 year)	52.2%(10 year)
	LRFS	87.5%	75%	72.9%(3 year)	90.0%(10 year)
	MFS	87.5%	53%	33.4%(3 year)	31.0%(10 year)
Recurrent localized STS	DSS	92.3%	33%	62.8%(OS, 3 year)	44.1%(10 year)
	LRFS	81.8%	85%	97.0%(3 year)	83.7%(10 year)
	MFS	82.1%	23%	57.2%(3 year)	42.2%(10 year)
Indications in primary localized STS^*^		^a^(22%), ^b^(78%)	^a^(39%), ^b^(61%)	^b^(13%)	^b^(50%), ^c^(50%)
Indication and timing in recurrent localized STS^*^		^a^(23%), ^b^(92%)	^a^(36%), ^b^(41%)	^b^(16%)	^a^(17.4%), ^b^(52.2%)

STS: soft tissue sarcoma, DSS: disease specific survival, LRFS: local recurrence free survival, MFS: metastasis free survival, NA: not available

^*^Regarding indications, alphabetics imply the followings; ^a^Significant impairment or loss of limb function due to tumor, ^b^No technical salvage options = extensive involvement and involvement of critical structure, ^c^Inoperable due to huge size

### Oncologic outcome: amputation vs. limb salvage surgery

**Fig. 2:**
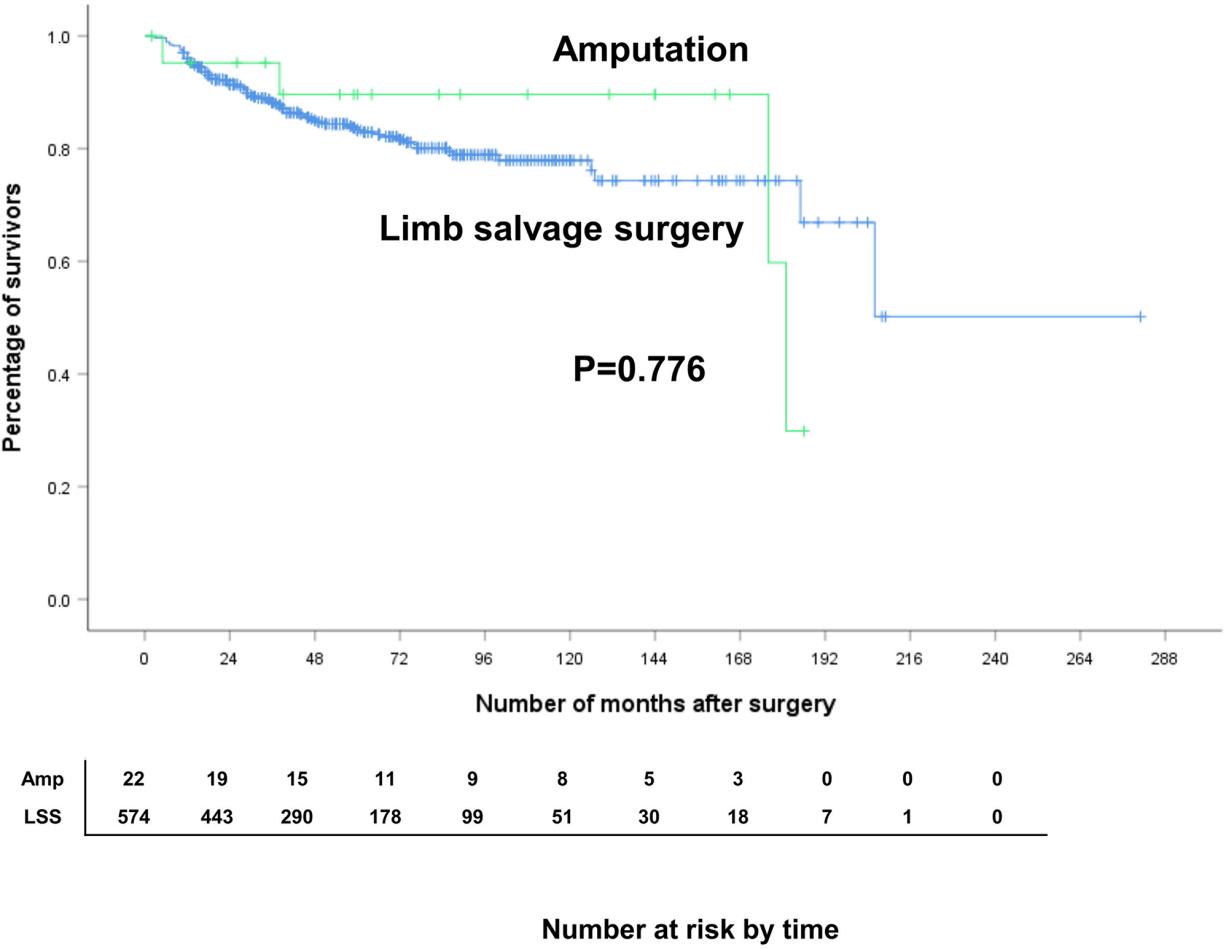
Disease specific survival of amputation group and limb salvage surgery group. (*P* = 0.146)

The oncologic outcome of localized disease was also compared between the amputation group (*n* = 22) and limb salvage group (*n* = 574). The 5-year DSS of the amputation group (89.6%) was not statistically different from that of the limb salvage group (83.3%) (*p* = 0.776) (Fig. 2). No significant difference was also found in the LRFS and MFS rates between the two groups.

## Discussion

In 1960s, amputation was recommended as the treatment for STS with high local recurrence rate [3]. LSS with multidisciplinary treatment has begun to replace amputation since 1970s [2, 14, 21]. However, amputation still sustains its role as a definitive treatment to achieve local control, and many studies have revisited its contemporary indication and outcome recently [13, 22–24]. Over the years, STS patients showed reluctance to undergo radical surgery such as amputation. The amputation rate for localized STS reported in our study was similar but slightly lower than that reported in Western studies, and the rate of amputation with primary curative intent was lower (1.8%). The DSS and MFS rates of amputation for localized disease reported in this study were higher than that indicated in previous studies, while the LRFS rates were similar.

Our study has some limitations. First, this study evaluated a small number of extremity STS patients from a single tertiary referral center, which might be subject to inherent selection bias and referral bias. In the subgroup analysis of the PL and RL groups, the analysis was inevitably descriptive without statistical confirmation due to the small sample size. Future multi-center study or national level study is necessary to re-evaluate or confirm our results. Secondly, due to its retrospective study design, it was difficult to assess the reason of low amputation rate in our institute. We admit that there might have been patients who needed amputation were treated with LSS and adjuvant treatments. We instead tried to overcome the limitation by analyzing the indications for amputation in the PL and RL group. We also compared the amputation rate of our cohort with those reported in the literature. On the other hand, we compared the oncologic outcomes between the amputation group and LSS group to assess whether this low rate of amputation might have failed in achieving proper local control. There might have been multiple factors influenced avoidance of amputations including preference of the surgeon, cultural issues such as the Confucianism. We believe these factors should be assessed by analyzing the patients who did not undergo amputation and underwent LSS with adjuvant treatment in the future.

Thirdly, our study could not analyze socioeconomic status (SES), which is important for health care accessibility. Some researchers suggest that SES is more predictive of treatment quality received than race or ethnicity [20, 25]. However, this element varies depending on countries and Korea has a universal health insurance system with short waiting times while accessibility to tertiary referral centers is high [26]. In addition, this study is a review of single institute. Therefore, consideration for this status is also necessary in future multi-center design or National level study.

The amputation rate for localized disease was 3.6% (22/596), while that with primary curative intent was 1.8% (9/490). Both of the values were similar or slightly lower than those indicated in previous Western studies (Table 2). In Europe, according to the most recent study of Royal Marsden Hospital with 556 STS patients, Smith et al. reported that the amputation rate with primary curative intent was 4.1% [13]. Keus et al. reported 8.3% of amputation rate from Netherland [27]. Trovik et al. reviewed 1,613 adult patients who were registered to the Scandinavian Sarcoma Group Registry and reported a localized amputation rate of 22.0% with a primary amputation rate of 9.6% [12]. In Canada, Ghert et al. reported a primary amputation rate of 6.3% in 413 patients [15]. In the United states, Williard et al. at the Memorial Sloan-Kettering Cancer Center reported 14.1% of amputation rate with 557 STS patients.[28]. Twenty years later, Alamanda et al. reviewed 278 patients with STS and reported a localized amputation rate of 5.7% and a primary amputation rate of 3.5% [29]. The amputation rate has significantly decreased over the years and the rate in our institute was similar or slightly lower than other references. These numbers also show that there are certain indications that the amputation is necessary.

In clinico-pathologic factors, the median tumor size was less than 5 cm and smaller than those of previous studies. Erstad et al. reported that 69% of amputated tumors were larger than 5 cm, while 34% were larger than 10 cm. Stevenson et al. reported median tumor sizes of 8.7 cm in the entire cohort and 11.5 cm in the primary localized group [22, 23]. The pathologic grades and depth in our cohort were similar with those in previous studies. Thus, we carefully assumed that less aggressive tumor biology with small size tumor might have contributed to the favorable oncologic outcome of our cohort. No difference was observed in the oncologic outcome between the PL group and RL group. Smith et al. reported the same phenomenon in his retrospective review of 69 patients who underwent amputation [13]. Therefore, Smaller tumors might have been the explanation for good prognosis of the patients with amputation. We believe there might have been other factors affected the patients with aggressive large sarcomas to betreated with LSS instead of amputation. This should be analyzed in the future study. When the PL and RL groups were compared, there were no difference in tumor size between the two groups. In Stevenson et al.’s recent study, the median tumor size of the primary amputation group (11.5 cm) was larger than that of the group who underwent amputation as non-primary treatment (6.1 cm) (*p* = 0.008). Therefore, the size was not the definitive factor for deciding amputation in our study. Erstad et al. classified the tumors located in the hand and foot as the distinctive group and suggested that this group had a better overall survival [22]. In our PL group, two patients had foot tumors and one had a hand tumor. All of them were successfully treated with amputation. Hand and foot tumors were not observed in the RL group. On histologic grade, grade 3 was the most common in the PL group (44.4%) and grade 2 was the most common in the RL group (61.5%). This result is similar to that reported in Smith et al.’s review of 69 STS patients treated with major amputation [13], but slightly different from that reported in Erstad et al’s review, in which grade 3 tumors were the most common histology in both groups. Even though grade 2 was more frequent in RL group, RL tumors are recurrent tumors which have survived the selective pressure of LSS and this point should be considered in treatment strategy [22]. Multifocality was a characteristic of RL tumors and statistically different from that of PL tumors (*p* = 0.031). Approximately 62% of RL tumors had multifocal location. Stojadinovic et al. suggested multiple local recurrence and multi-compartment disease as principal reasons for amputation [30]. Even a single lesion can involve multiple compartments. Multifocal recurrent lesions usually involve multiple compartments (100% in the RL group) and entail inevitable functional loss in an attempt to achieve R0 margin. Therefore, multifocality of recurrent STS can be considered as a distinctive factor for considering amputation.

\In relation to the oncologic outcomes, the DSS and MFS rates in our study were higher than those in previous studies, while the LRFS rate was compatible with that in Western studies (Table 4). Most previous studies reported DSS or OS rates of 40–50% with similar MFS rates. Although the mode of surgery is not a predictive factor for metastasis, tumors requiring amputation tend to have aggressive biology such as larger size, higher grade, and deep location in literatures. [31–33]. Japanese report of Tokai musculoskeletal consortium with 55 patients showed 52.8% of DSS and 63.1% with 42 patients without metastasis at presentation [18]. In this study, they have excluded amputations distal to wrist and ankle which might have influence the results while there were 3 amputations distal to wrist and ankle in our study.

## Conclusion

The amputation rate of extremity STS in our institute in East Asia was similar but slightly lower than that reported in Western studies. The oncologic outcome of amputation reported in this study was higher than that indicated in Western studies and oncologic outcome of amputation was not statistically different from those of limb salvage surgery. However, considering the small cohort in single institute study, there is a possibility of inevitable selection bias and future multi-center study is necessary. From our results, amputation is still a feasible option for appropriately selected patients unsuitable for limb-conserving surgery.

## Data Availability

All data generated or analysed during this study are included in this published article.

## References

[1] Eilber FR, Mirra JJ, Grant TT, Weisenburger T, Morton DL (1980). Is amputation necessary for sarcomas? A seven-year experience with limb salvage. Ann Surg.

[2] Suit HD, Russell WO, Martin RG (1973). Management of patients with sarcoma of soft tissue in an extremity. Cancer.

[3] Cantin J, McNeer GP, Chu FC, Booher RJ (1968). The problem of local recurrence after treatment of soft tissue sarcoma. Ann Surg.

[4] Pisters PW, Pollock RE, Lewis VO, Yasko AW, Cormier JN, Respondek PM, Feig BW, Hunt KK, Lin PP, Zagars G (2007). Long-term results of prospective trial of surgery alone with selective use of radiation for patients with T1 extremity and trunk soft tissue sarcomas. Ann Surg.

[5] Rosenberg SA, Tepper J, Glatstein E, Costa J, Baker A, Brennan M, DeMoss EV, Seipp C, Sindelar WF, Sugarbaker P (1982). The treatment of soft-tissue sarcomas of the extremities: prospective randomized evaluations of (1) limb-sparing surgery plus radiation therapy compared with amputation and (2) the role of adjuvant chemotherapy. Ann Surg.

[6] Williard WC, Hajdu SI, Casper ES, Brennan MF (1992). Comparison of amputation with limb-sparing operations for adult soft tissue sarcoma of the extremity. Ann Surg.

[7] Bickels J, Wittig JC, Kollender Y, Kellar-Graney K, Malawer MM, Meller I (2002). Sciatic nerve resection: is that truly an indication for amputation?. Clin Orthop Relat Res.

[8] Clark MA, Thomas JM (2003). Amputation for soft-tissue sarcoma. Lancet Oncol.

[9] Ferrone ML, Raut CP (2012). Modern surgical therapy: limb salvage and the role of amputation for extremity soft-tissue sarcomas. Surg Oncol Clin N Am.

[10] Henshaw RM, Priebat DA, Perry DJ, Shmookler BM, Malawer MM (2001). Survival after induction chemotherapy and surgical resection for high-grade soft tissue sarcoma. Is radiation necessary?. Ann Surg Oncol.

[11] Pitcher ME, Fish S, Thomas JM (1994). Management of soft tissue sarcoma. Br J Surg.

[12] Trovik CS, Scanadinavian Sarcoma Group P (2001). Local recurrence of soft tissue sarcoma. A scandinavian Sarcoma Group Project. Acta Orthop Scand Suppl.

[13] Smith HG, Thomas JM, Smith MJF, Hayes AJ, Strauss DC (2018). Major amputations for extremity Soft-Tissue Sarcoma. Ann Surg Oncol.

[14] Morton DL, Eilber FR, Townsend CM, Grant TT, Mirra J, Weisenburger TH (1976). Limb salvage from a multidisciplinary treatment approach for skeletal and soft tissue sarcomas of the extremity. Ann Surg.

[15] Ghert MA, Abudu A, Driver N, Davis AM, Griffin AM, Pearce D, White L, O’Sullivan B, Catton CN, Bell RS (2005). The indications for and the prognostic significance of amputation as the primary surgical procedure for localized soft tissue sarcoma of the extremity. Ann Surg Oncol.

[16] Mann GN (2005). Less is (usually) more: when is amputation appropriate for treatment of extremity soft tissue sarcoma?. Ann Surg Oncol.

[17] Ngan R, Wang E, Porter D, Desai J, Prayogo N, Devi B, Quek R (2013). Soft-tissue sarcomas in the Asia-Pacific region: a systematic review. Asian Pac J Cancer Prev.

[18] Hagi T, Nakamura T, Nagano A, Koike H, Yamada K, Aiba H, Fujihara N, Wasa J, Asanuma K, Kozawa E (2022). Clinical outcome in patients who underwent amputation due to extremity soft tissue sarcoma: Tokai Musculoskeletal Oncology Consortium study. Jpn J Clin Oncol.

[19] Lazarides AL, Visgauss JD, Nussbaum DP, Green CL, Blazer DG, Brigman BE, Eward WC (2018). Race is an independent predictor of survival in patients with soft tissue sarcoma of the extremities. BMC Cancer.

[20] Martinez SR, Robbins AS, Meyers FJ, Bold RJ, Khatri VP, Goodnight JE (2008). Racial and ethnic differences in treatment and survival among adults with primary extremity soft-tissue sarcoma. Cancer.

[21] Rosenberg SA, Kent H, Costa J, Webber BL, Young R, Chabner B, Baker AR, Brennan MF, Chretien PB, Cohen MH (1978). Prospective randomized evaluation of the role of limb-sparing surgery, radiation therapy, and adjuvant chemoimmunotherapy in the treatment of adult soft-tissue sarcomas. Surgery.

[22] Erstad DJ, Ready J, Abraham J, Ferrone ML, Bertagnolli MM, Baldini EH, Raut CP (2018). Amputation for Extremity Sarcoma: contemporary indications and outcomes. Ann Surg Oncol.

[23] Stevenson MG, Musters AH, Geertzen JHB, van Leeuwen BL, Hoekstra HJ, Been LB (2018). Amputations for extremity soft tissue sarcoma in an era of limb salvage treatment: local control and survival. J Surg Oncol.

[24] van Houdt WJ, Griffin AM, Wunder JS, Ferguson PC (2018). Oncologic outcome and quality of Life after Hindquarter Amputation for Sarcoma: is it worth it?. Ann Surg Oncol.

[25] McGory ML, Zingmond DS, Sekeris E, Bastani R, Ko CY (2006). A patient’s race/ethnicity does not explain the underuse of appropriate adjuvant therapy in colorectal cancer. Dis Colon Rectum.

[26] Song YJ (2009). The South Korean health care system. Jmaj.

[27] Keus RB, Rutgers EJ, Ho GH, Gortzak E, Albus-Lutter CE, Hart AA (1994). Limb-sparing therapy of extremity soft tissue sarcomas: treatment outcome and long-term functional results. Eur J Cancer.

[28] Williard WC, Collin C, Casper ES, Hajdu SI, Brennan MF (1992). The changing role of amputation for soft tissue sarcoma of the extremity in adults. Surg Gynecol Obstet.

[29] Alamanda VK, Crosby SN, Archer KR, Song Y, Schwartz HS, Holt GE (2012). Amputation for extremity soft tissue sarcoma does not increase overall survival: a retrospective cohort study. Eur J Surg Oncol.

[30] Stojadinovic A, Jaques DP, Leung DH, Healey JH, Brennan MF (2001). Amputation for recurrent soft tissue sarcoma of the extremity: indications and outcome. Ann Surg Oncol.

[31] Collin C, Godbold J, Hajdu S, Brennan M (1987). Localized extremity soft tissue sarcoma: an analysis of factors affecting survival. J Clin Oncol.

[32] Mann GB, Lewis JJ, Brennan MF (1999). Adult soft tissue sarcoma. Aust N Z J Surg.

[33] Shiu MH, Castro EB, Hajdu SI, Fortner JG (1975). Surgical treatment of 297 soft tissue sarcomas of the lower extremity. Ann Surg.

